# Oviductal extracellular vesicles interact with the spermatozoon’s head and mid-piece and improves its motility and fertilizing ability in the domestic cat

**DOI:** 10.1038/s41598-019-45857-x

**Published:** 2019-07-01

**Authors:** M. de A. M. M. Ferraz, A. Carothers, R. Dahal, M. J. Noonan, N. Songsasen

**Affiliations:** 1 Center for Species Survival, Smithsonian National Zoo and Conservation Biology Institute, 1500 Remount Road, Front Royal, Virginia 22630 USA; 2 Smithsonian National Zoo and Conservation Biology Institute, 1500 Remount Road, Front Royal, Virginia 22630 USA; 3 Department of Biology, University of Maryland, College Park, Maryland 20742 USA

**Keywords:** Embryology, Extracellular signalling molecules

## Abstract

Fertilization and early embryo development are regulated by a unique maternal-gamete/embryo cross-talk within the oviduct. Recent studies have shown that extracellular vesicles (EVs) within the oviduct play important roles in mediating this developmental process. Here, we examined the influence of oviductal EVs on sperm function in the domestic cat. We demonstrated that (1) EVs are enriched in proteins related to energy metabolism, membrane modification, and reproductive function; (2) EVs bound and fused with the membranes of the acrosome and mid piece; and (3) incubating sperm with EVs improved motility, fertilizing capacity of cat spermatozoa and prevented acrosomal exocytosis *in vitro*. These findings indicated that oviductal EVs mediate sperm function and fertilization in the cat and provides new insights to improve sperm cryopreservation and *in vitro* fertilization in the domestic and wild felids and human.

## Introduction

Due to the loss of natural habitat^1^, nearly half of wild felid species are listed as threatened by the International Union for Conservation of Nature (IUCN, 2018)^2^. Many of these species are kept in zoos and breeding centers for public awareness, research purposes and/or as security populations^3^, but successful management of small populations is threatened by losses in genetic diversity due to poor reproduction and/or health. Assisted reproductive technologies (ARTs), including artificial insemination and *in vitro* fertilization (IVF) have been applied to retain gene diversity and avoid inbreeding depression^4^. Although live offspring have been produced by IVF in a number of wild felids, including the Indian desert cat, African wildcat, serval, caracal, fishing cat, black-footed cat, tiger and the sand cat^5^, this technology has not been widely used in breeding programs, partly due to limited information on species-specific reproductive endocrinology, gamete biology and embryogenesis^6^. Furthermore, live birth rates from *in vitro* derived embryos is low in these species, varying from 0 to 40%^7^. Therefore, there is a critical need to establish improved *in vitro* conditions supportive of normal fertilization and embryonic development.

The oviduct (or fallopian tube in the human) provides the physiological environment that is essential for sperm storage and capacitation^8–15^, fertilization^11,16–18^ and embryo development^19–22^. After mating, sperm travel into the uterus, enter the oviduct and attach to the epithelium of the isthmus, a process which is necessary for extending sperm viability within the female reproductive tract^23^. Within the isthmus, sperm acquire hyperactivated motility and the ability to fertilize an oocyte (a process known as ‘capacitation’)^12^. It has been demonstrated that the oviduct facilitates gamete function and fertilization by the secretion of extracellular vesicles (EVs)^21,24,25^. EVs are membrane encapsulated particles containing regulatory molecules that contribute to cell-cell communication by carrying proteins, peptides, RNA species (microRNAs, mRNAs, and long non-coding RNAs), lipids, and DNA fragments^26–28^. EVs have been isolated from prostate, epididymal, uterine, follicular and oviductal fluids^21,24,29–36^. Yet, only a hand full of studies have investigated the role of oviductal EVs on gamete maturation and embryo production^21,24,32,37–39^. Previous studies have shown that the presence of oviductal EVs during embryo culture increases blastocyst rate and improves embryo quality during *in vitro* incubation in cattle^21^. Incubation of mouse sperm with oviductal fluid or exosomes increases a Ca^2+^ efflux pump protein, PMCA4, which is important for the acquisition of hyperactivated motility and fertilizing ability^24^. Recently, it also has been shown that oviductal EVs supplemented in embryo transfer medium increased birth rates after transferring *in vitro* derived embryos in the mouse^38^.

To date, there are no studies about the effects of oviductal EVs on sperm function and its fertilizing ability *in vitro*. Because of its importance as a biomedical model and for the applicability of these data to wild felids^4,40–42^ and humans^40^, we isolated cat oviductal EVs and investigated their protein content, interaction with sperm and influence on sperm function.

## Results and Discussion

### Exosomes are the most abundant EVs in the cat oviduct

**Figure 1:**
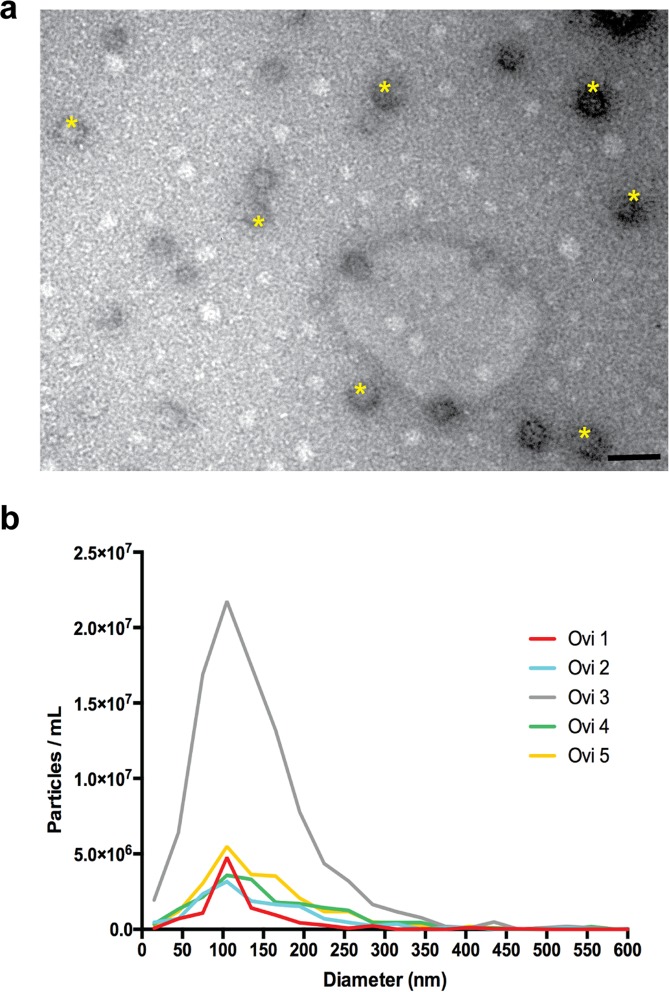
Transmission electron microscopy (TEM) and size distribution of cat oviductal EVS. (**a**) TEM image of oviductal EVs (stars); scale bar depicts 200 nm. (**b**) Size distribution of oviductal EVs measured by nanoparticle tracking analysis using the ZetaView. EVs were collected from five cats (Ovi1-5) the quadratic interpolation of the mean number of particles isolated from the oviducts of each individual donor cat is represented in the graph.

Previous studies have developed methods that allow consistent yield of high quality and quantity EVs^26,43,44^. In this study, we used Total Exosome Isolation Kit (Invitrogen, USA) to recover EVs from cat oviductal fluid. This method was shown to be superior to commonly used ultracentrifugation protocols in terms of EVs recovery yield, without changes in EVs quality^44^. Transmission electron microscopy (TEM) analysis showed the presence of EVs with sizes ranging from 40–150 ηm (Fig. 1a), and ZetaView nanoparticle tracking analysis (NTA) confirmed an average EV size of 129.7 ± 89.4 ηm. Overall, EV concentrations were similar among cats, except for one donor (*Ovi EVs 3*), from which higher EV levels were recovered (Table 1). The concentration of EVs ranged from 1.4 × 10^9^ to 8.6 × 10^9^ particles mL^−1^ (Fig. 1b and Table 1). Statistical analysis (ANOVA, F(4) = 33.474, p = 0.01, n = 5) revealed that there were significant differences in the concentration of EVs collected among donor cats and post hoc evaluations showed that the level observed in cat 3 was significantly different from cats 2, 4 and 5 (Tukey test p = 0.022, 0.03 and 0.048, respectively). Although there are large variations in EV size, (15–895 ηm), the majority (74%) of EVs were 40–150 ηm in diameter which is typical for exosomes^44^ (Fig. 1b and Table 1).

**Table 1:** Nanoparticle tracking analysis of oviductal extracellular vesicles isolated from five cats.

Sample ID	Cat age (months)	Diameter (ηm) (mean ± SEM)	Exosomes (% of 40–150 ηm)	Total Particles (per mL)
*Ovi EVs 1*	24	129.9 ± 67.2	85.3	3.76 ± 1.49 × 10^9a,b^
*Ovi EVs 2*	18	129.8 ± 103.5	69.2	1.43 ± 1.15 × 10^9a^
*Ovi EVs 3*	7	120.8 ± 85.7	77.3	8.63 ± 2.12 × 10^9b^
*Ovi EVs 4*	8	137.0 ± 90.9	64.8	1.77 ± 0.34 × 10^9a^
*Ovi EVs 5*	12	130.9 ± 99.7	73.2	2.09 ± 0.96 × 10^9a^

Letters^(a,b)^ indicates significant differences among cats (p < 0.05).

It’s known that steroids hormones influence the oviductal environment, promoting changes on its transcriptome and proteome, both *in vivo* and *in vitro*^45–47^. EVs were previously identified in the bovine oviductal fluid at different stages of the estrous cycle, and demonstrated that their composition is also under hormonal regulation^25^. For bovine, RNA-sequencing identified 903 differentially expressed transcripts in EVs across the estrous cycle^25^. Moreover, from the 336 clusters of EVs proteins, 170 were differentially abundant among stages of the estrous cycle^25^. In the present study, we collected oviductal EVs from cats during the follicular phase of reproductive cycle (neither corpora hemorrhagica nor corpora lutea was present). Therefore, it is unlikely that the high EV particles observed in cat 3 was due to a variation in reproductive stage. Trauma or inflammation can increase the number of EVs secreted in the plasma^48^, likewise, the increased EV number in cat 3 might have been due to an injury or pathology that was not visibly detected during oviduct flushing.

### Oviductal EVs protein content

A total of 1,511 protein groups were identified, out of 4,879 protein entries (Supplementary Data 1). The numbers of proteins identified in cat EVs were more than three-times higher than the amount previously observed in bovine oviductal exosomes and fluids (315 and 482 total proteins, respectively)^21,46^, but less than the level reported in the human (5,177 total proteins)^49^. The difference in the amount of proteins between bovine and cat oviductal EVs may be due to the mass spectrometry method used in the two studies. In the present study, oviductal EVs were analyzed using ultraperformance liquid chromatography and tandem mass spectrometry (UPLC-MS/MS). In the bovine study, the samples were analyzed by SDS-PAGE combined with nanoLC–MS/MS. It has been shown that improved sensitivity and efficiency of UPLC-MS/MS increases the ability to detect different proteins isoforms, with higher quantification limits^50^. Moreover, a single-pot, solid-phase-enhanced sample preparation (SP3) for protein isolation was used, which has been shown to have a high recovery of protein input^51^.

In the cat oviductal EVs, specific EVs markers, such as transmembrane- or lipid-bound extracellular proteins (CD9, CD63, CD109, CADM1, ITGA3, ITGA6, ITGB1, ITGB3, ITGB4, ESP8, and MFGE8), and cytosolic proteins (TSG101, ANXA1-7, ANXA11, RAB1A, RAB1B, RAB2A, RAB5B, RAB5C, RAB6A, RAB6B, RAB10, RAB11A, RAB11B, EEA1, and SDCBP) were detected, indicating the EV origin^43^ of the samples analyzed. Furthermore, two functionally grouped gene ontology (GO) cellular component pathways related to EVs were identified: (1) extracellular exosome (GO:0070062) and (2) extracellular vesicle (GO:1903561); these pathways had 709 and 713 total proteins respectively, with a fold enrichment of 2.64 each.

**Figure 2:**
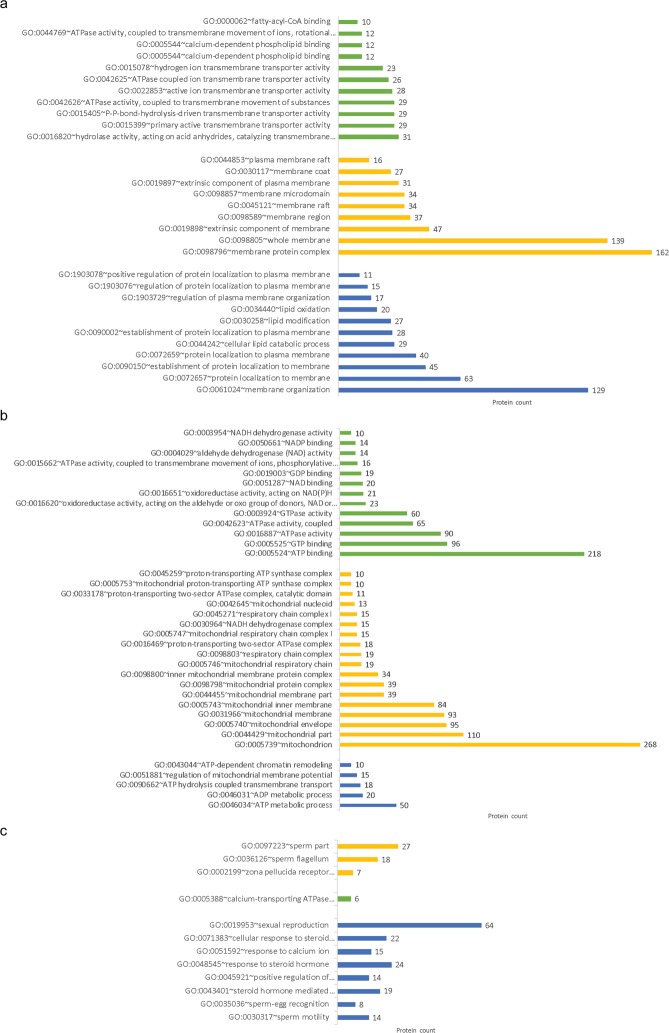
GO pathways related to the potential role of oviductal EVs on sperm characteristics and fertilizing ability. GO molecular function (green), cellular components (yellow) and biological process (blue) pathways related to (**a**) membrane modification, (**b**) energy metabolism, and (**c**) reproductive function.

Functional analysis using PANTHER database revealed that the identified proteins were part of different GO biological processes, including metabolism (30.2%), cellular component organization (18.5%), biological regulation (17.1%), localization (15.1%), cellular function (10.3%), response to a stimulus (4.7%), multicellular organism process (3.9%), development (3.6%), biological adhesion (2.5%), signaling (1.3%), immune system (1.1%), reproduction (0.7%), locomotion (0.4%), multi organism process (0.2%), and growth (0.1%). These pathways were similar to the ones identified in bovine oviductal EVs proteins^21^. Related to the potential role of oviductal EVs on sperm characteristics and fertilizing ability, three main clusters of GO molecular function, cellular components and biological process pathways were recognized by DAVID database: These include pathways related to (1) energy metabolism (Fig. 2a); (2) membrane modification (Fig. 2b); and (3) reproductive function (Fig. 2c). Supplementary Data 2 shows all GO molecular functions, cellular components and biological process pathways identified.

Proteins related to sperm motility (ATP1A4, ATP2B4, DNAJA1, APOB, CCDC39, CCDC40, DPCD, DNAH1, DNAH5, NPHP4, PGK2, PLTP, TEKT2, and TEKT3), sperm-egg recognition (NME5, NME7, NME9, NME1, CAD, MTOR, and UPRT), and sperm binding to zona pellucida (CCT2, CCT3, CCT4, CCT5, CCT7, CCT8, OVGP1, and TCP1) were included in the functionally grouped GO pathways determined by DAVID. A diagram of proteins and their network pathways can be seen in Supplementary Fig. 1.

### Oviductal EVs bind to sperm acrosome and mid-piece

**Figure 3:**
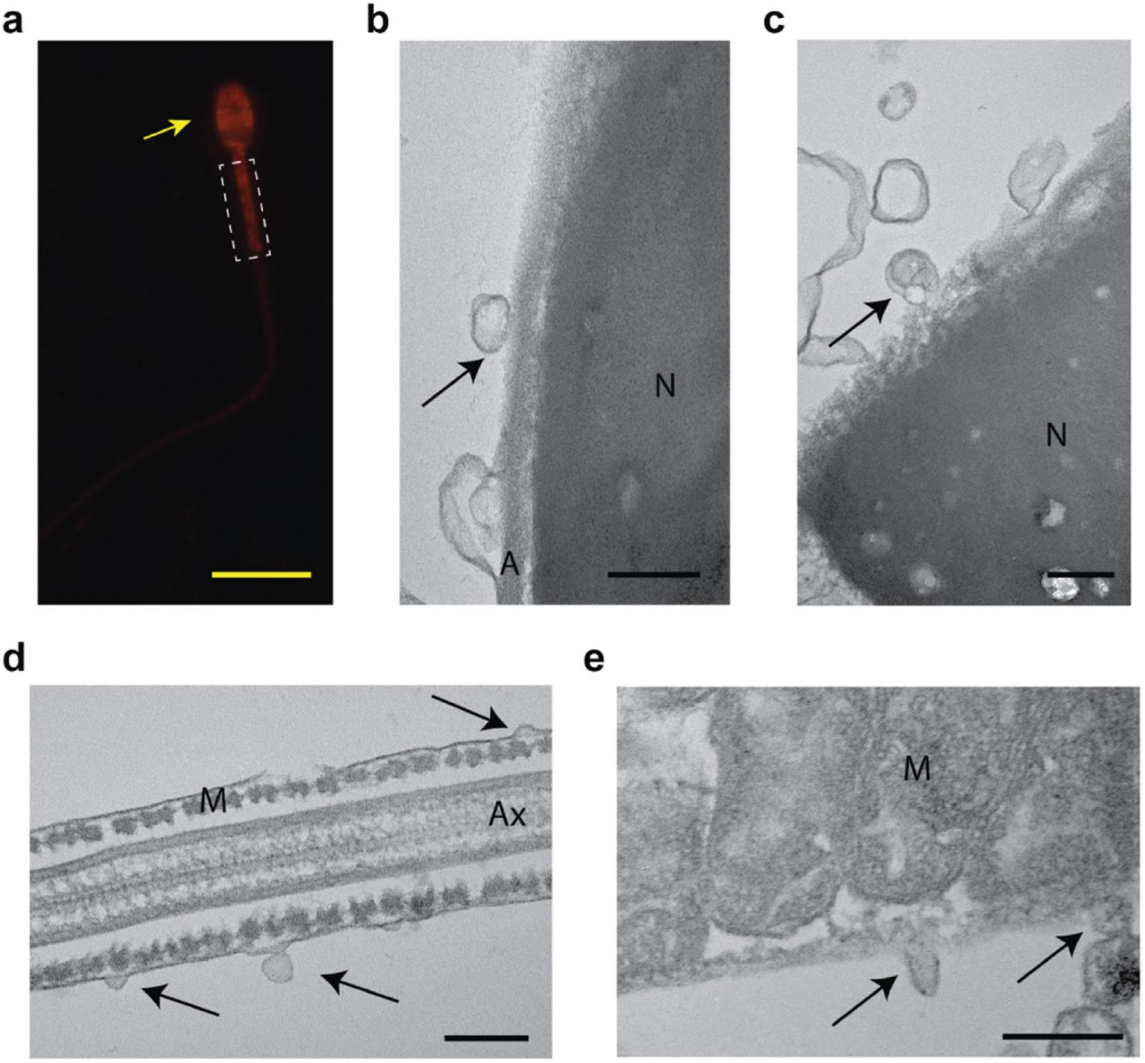
Uptake of oviductal EVs by spermatozoa. (**a**) Sperm incubated with BODIPY^®^ TR Ceramide labeled oviductal EVs, note that EVs bind to sperm acrosome (arrow) and mid-piece (dashed box). (**b**–**e**) TEM images of sperm incubated with EVs (black arrows), in (**b**,**c**) EVs bound to sperm head, showing nucleus (N) and acrosome (A); and on (**d**,**e**) EVs bound to sperm mid-piece, showing axoneme (Ax) and mitochondria (M). Scale bar: yellow = 15 μm, black = 200 ηm.

Small EVs (40–150 ηm; exosomes) facilitate the transportation of proteins, microRNAs and other factors from luminal fluids to the sperm surface^24,34–36,52^. We used fluorescence microscopy and TEM to investigate the interaction between oviductal EVs and cat spermatozoa. Incubating cat epididymal sperm with BODIPY^®^ TR Ceramide labeled EVs (1:2 v/v sperm to EVs, n = 3) for 1 hour resulted in a higher percentage of EVs bound spermatozoa (>96%) than samples incubated for 15 (64%) and 30 (84%) minutes (paired sample t-test, p = 0.02 and 0.04, respectively). Cat oviductal EVs bound to the acrosomal region of the sperm head and mid-piece (Fig. 3a). TEM confirmed the fusion of 60 to 180 ηm EVs to the outer acrosomal membrane and mid piece (n = 2, Fig. 3b–e). Bound vesicles were not detected in samples analyzed in the absence of oviductal EVs. This binding pattern is similar to that described previously for mouse sperm incubated with oviductal, epididymal and uterine EVs^32,53^.

### Incubation with oviductal EVs increased sperm motility and sustained acrosomal integrity

**Figure 4:**
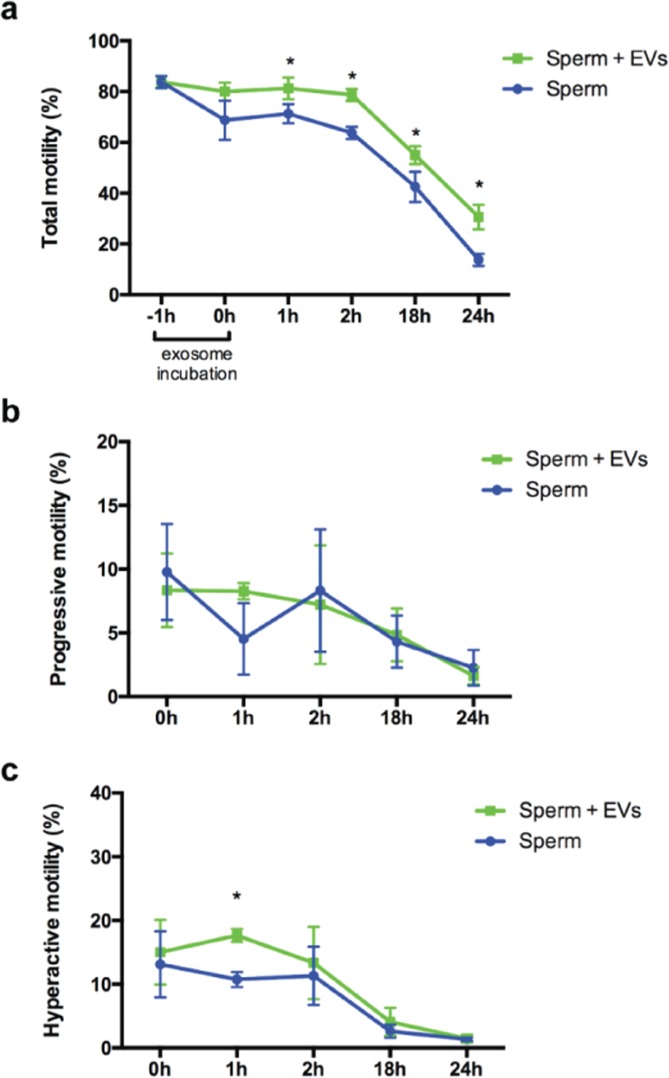
Total (**a**), progressive (**b**) and hyperactive (**c**) motility of sperm incubated with or without oviductal extracellular vesicles (EVs) at 0, 1, 2, 18 and 24 hours. Symbol indicates statistically significant difference (p < 0.05, paired samples t-test) for sperm + EVs vs Sperm for each time point (n = 5).

The functionality of a spermatozoon generally depends on its (1) progressive motility^54^, (2) acrosomal integrity^55^, and (3) the ability to undergo capacitation and acrosomal exocytosis^56^. It is known that the oviductal epithelium influences sperm function as well as supports gamete interaction and early embryo development^9,12,18,57–60^. A previous study has shown that human sperm sustain motility longer when they were incubated with homologous oviductal epithelium than those without co-incubation^61^. Here, we investigated whether oviductal EVs could sustain cat sperm motility *in vitro*. Sperm were incubated for 1 h with EVs collected from cat oviducts (n = 5 cats). Incubation of sperm with EVs sustained a greater percentage of motile sperm (paired sample t-test, p = 0.016, 0.000, 0.030, and 0.018, for 1, 2, 18 and 24 h, respectively; n = 5; Fig. 4a) than those without EVs, and this trend was maintained throughout the 24-hour incubation. These results are in agreement with previous studies in which incubating bovine sperm with oviductal fluid improved sperm motility for as long as 6 hours^9^. Incubation of cat spermatozoa with oviductal EVs did not affect progressive motility (Fig. 4b) and only increased hyperactive motility at 1 h post-incubation (paired sample t-test, p = 0.007; Fig. 4c).

Sperm acquires energy from molecules present in the seminal plasma and in the female reproductive tract^62^. As in other mammalian cells, most of this energy is transformed into adenosine tri-phosphate (ATP), which is critical for flagella movement, membrane fusion events during the acrosome reaction, and transportation of ions and other molecules through the sperm membranes^62^. We demonstrated that, similar to bovine^21^, cat oviductal exosomes carry proteins related to energy metabolism, such as ATP synthase, V-type proton ATPase subunit E, ATP binding, plasma membrane calcium-transporting ATPase 2, ADP/ATP translocase 1, mitochondrial ATP synthase, cytochrome c oxidase, acyl-CoA dehydrogenase, succinate-CoA ligase [ADP/GDP-forming] subunit alpha, NADPH, ATPase Na^+^/K^+^ transporting, ATPase plasma membrane Ca^2+^ transporting, among others. Because energy production is essential for sperm flagella movement, we speculated that these proteins from cat oviductal EVs can be delivered to the sperm mid-piece and could explain the higher sperm motility observed. Moreover, HSP70, OVGP1 and different apolipoproteins (APOA1, APOB, APOE) were also present in the EVs, which were previously reported to be positively correlated with sperm motility in bulls and human^63–65^. Cumulative findings from our and previous studies suggests that HSP70, OVGP1 and apolipoproteins may also contribute to the higher motility observed in the cat sperm incubated with oviductal EVs.

**Figure 5:**
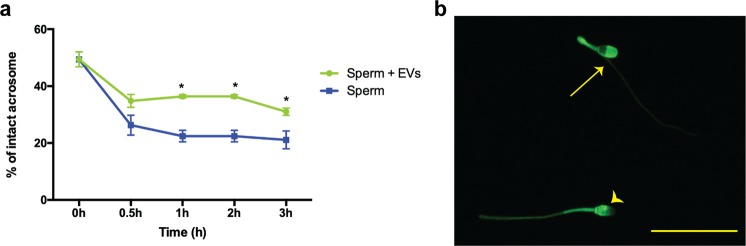
(**a**) Percentages of sperm with intact acrosome in samples incubated with or without oviductal EVs for 0, 0.5,1, 2 and 3 hours. There were statistically significant differences (paired samples t-test) between the two treatments at 1, 2 and 3 h (p = 0.0001, 0.0001 and 0.0188, respectively; n = 5). In (**b**), sperm with an intact (arrow) or non-intact (arrow heads) acrosome stained with FITC-PNA. Scale bar = 20 μm.

Another requisite event for fertilization in mammals is the ability of spermatozoa to undergo acrosomal exocytosis (AE)^66^. AE starts during the sperm transit through the oviduct, and is modulated by the oviductal and follicular fluids, cumulus oophorous cells and the zona pellucida^9,55,66^. In contrast to the previous reports in cows and human, in which oviductal fluid induced AE^9,66^, incubating cat sperm with oviductal EVs prevented *in vitro* acrosome exocytosis of spermatozoa incubated under a non-capacitation condition. The percentages of cat sperm with intact acrosome after 30 min incubation in PBS were 34 ± 3.1 and 23 ± 4.7% for samples incubated with and without EVs, respectively, both of which were less than 49 ± 3.7% observed at 0 hour incubation (p = 0.0041 and 0.0003, respectively; n = 5, paired samples t-test) (Fig. 5). Extending the incubation period to 1 hour significantly decreased the percentage of sperm with intact acrosome in the absence of EVs (P = 0.03), whereas acrosome integrity of EVs exposure samples remained constant throughout the culture period. We have shown in the earlier experiment that the ability of EVs to bind to sperm plasma membrane is time dependent with maximum binding (96%) observed at 1 hour incubation. Therefore, the reduction in acrosome integrity in the presence of EVs during the first 30 min is likely due to insufficient time for EVs to bind to the sperm plasma membrane to fully exert their protective effect. The domestic cat is an induced ovulator, with ovulation occurring within 25–50 h after mating^67^. As early as 30 min after mating, spermatozoa can be observed in the oviduct of the cat^68^. Cat sperm must maintain acrosomal integrity for 25–50 h within the female reproductive tract for them to successfully fertilize an oocyte. Therefore, we hypothesize that oviductal EVs, especially during the preovulatory period, play roles in preventing sperm from undergoing acrosomal exocytosis prematurely.

Steps for capacitation, including acquisition of hyperactive motility and acrosome reaction, are also dependent on the lipid composition of the sperm membrane and its interaction with membrane proteins^69^. Oviductal EVs were rich in proteins that are part of important membrane modification processes, including, but not limited to, regulation of protein localization to plasma membrane (GO:1903076), membrane raft organization (GO:0031579), and regulation of cholesterol metabolic process (GO:0090181), which could also influence the differences observed in sperm function. Furthermore, sperm membrane lipid composition is also correlated with survival success after cryopreservation^70^. Significant sperm membrane lipid changes are associated with freezing/thawing procedures^71^. Incubating sperm with oviductal EVs, or specific EVs factors, prior to cryopreservation or during thawing procedure could influence the sperm membrane lipids composition and improve its functionality post-thawing.

### Oviductal EVs improve sperm’s fertilizing ability

**Figure 6:**
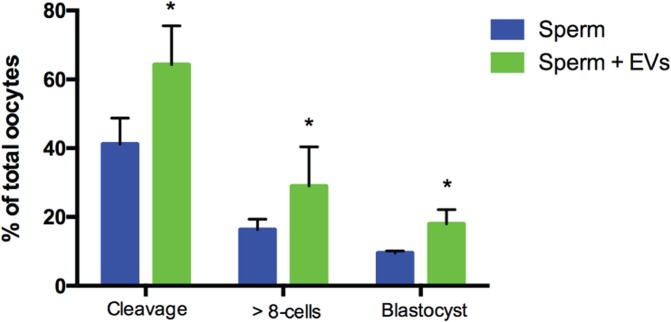
Effect of sperm incubation with (Sperm + EVs) or without (Sperm) oviductal extracellular vesicles (EVs) on fertilization rate (cleavage), percentage of embryos of 8 or more cells (>8-cells) and blastocyst rate (blastocyst). Symbol indicates statistically significant difference (General Linear Model, after correction for male effect) for sperm + EVs *vs* Sperm (p = 0.0013, 0.027 and 0.014 for cleavage, >8 cells and blastocyst respectively).

Here we examined the effect of cat oviductal EVs on IVF and embryo development. Cumulus-oocyte complexes (COCs) were inseminated with fresh spermatozoa that was previously incubated for 1 h with or without oviductal EVs. Our data revealed that incubating cat sperm with EVs increased cleavage rate by 23% compared to the non-EV samples (General linear model - GLM, p = 0.013; Fig. 6). As a consequence of a higher cleavage rate, a 12% increase in the number of >8 cell-embryos at Day 4 and an 8% increase in blastocyst rates were observed (GLM, p = 0.027 and p = 0.014, respectively). Cat oviductal exosomes contain proteins known to be important for fertilization, such as OVGP1 (improves sperm viability and motility *in vitro*)^65^, CD9, TCP1, CCT3, CCT4, CCT7, CCT8, (important for sperm-oocyte binding)^72,73^, HSP70 (improves sperm survival *in vitro*)^74^ and HSP90 (mediates sperm hyperactivation and acrosome exocytosis)^21,75^. The transfer of these proteins from the oviductal EVs to the spermatozoa could explain the increased fertilization rates observed.

Another known benefit of the oviductal environment is the reduction of polyspermic fertilization^18,76,77^. In this study, no difference in polyspermy was detected between samples incubated with oviductal EVs and no-EVs control (17 ± 9 vs 11 ± 5%, *sperm* and *sperm* + *EVs* respectively; GLM, p = 0.097). The incidence of polyspermy in cats is similar to that seen in the human (ranging from 3 to 30%) but it is lower than the rate observed in the pig (ranging from 14 to 93%)^76^. Our results suggest that the prevention of polyspermy in the cat likely depends on the effects of the oviductal environment on the oocyte. Indeed, incubating oocytes with oviductal fluid or its specific protein, the oviductal glycoprotein 1 (OVGP1) reduced polyspermy rates in the pig^77,78^. Recently, it was also shown that the use of a 3D culture system that mimics the oviductal microenvironment during IVF, significantly reduces polyspermy rates in the cow^18^. Future studies should examine the effect of incubating oocytes with oviductal EVs on the incidence of polyspermic fertilization in the domestic cat.

## Conclusions

This study showed that exosomes were the most abundant oviductal EVs in cats, and that they bind to the acrosomal region of the sperm head as well as the mid-piece. We also demonstrated that incubating sperm with EVs enhanced and sustained motility and acrosome integrity as well as improved fertilizing ability of cat epididymal sperm. The increased motility could be due to the transfer of important proteins from EVs to sperm, such as HSP70, OVGP1, apolipoproteins and proteins related to energy metabolism. Furthermore, increased fertilization was likely due to proteins related to sperm-oocyte recognition and binding, such as CD9, TCP1, and CCTs. The use of oviductal EVs *in vitro* has the potential to improve ART results for domestic and wild felids especially in individuals/species exhibiting poor seminal characteristics. Moreover, the findings serve as the foundation for future studies aimed at identifying oviductal factors that enhance sperm survival and fertilizing ability. Such studies offer promising avenues for the development of strategies for long-term sperm preservation and for designing improved *in vitro* culture conditions for IVF and embryo development.

## Methods

All male and female reproductive tracts were opportunistically collected from local veterinary clinics after routine neutering and spaying procedures of household and stray cats. No additional permissions were required since these biological materials were designated for disposal via incineration.

### Oviductal extracellular vesicles (EVs) isolation

Oviducts of domestic cats (7 months to 4 years old, n = 16) in follicular phase (ovaries without the presence of corpora hemorragica and/or lutea;) were recovered after routine ovariohysterectomy at local veterinary clinics and transported (at 4 °C) to the laboratory within 6 h of excision. After being washed three times in Phosphate Buffer Saline solution (PBS, GIBCO, USA), a 23G needle was inserted through the fimbria opening and the entire oviduct was flushed with 1 ml of PBS. The flush was centrifuged at 2,000 × g (room temperature) for 30 minutes to remove cells and debris, then the supernatant was mixed with 500 μL of the Total Exosome Isolation Reagent (Invitrogen, USA) and incubated overnight at 4 °C. The samples were centrifuged at 10,000 × g for 1 hour, and the pellet resuspended in 50 μL of PBS. EVs were then purified by Exosome Spin Columns (Invitrogen, USA), following manufacturer’s instructions and kept at −20 °C until use.

### Oviductal EVs quantification

Nanoparticle tracking analysis was done using the ZetaView S/N 17–332 (Particle Metrix, Meerbusch, Germany) and data analyzed using its software (ZetaView 8.04.02) by Alpha Nano Tech (Research Triangle Drive, NC, USA) as previously described^44^. For each donor cat (n = 5), oviductal EVs sample (1 ml) was diluted 100X in PBS, loaded into the cell, and the instrument measured each sample at 11 different positions throughout the cell, with three cycles of readings at each position. The pre-acquisition parameters were: sensitivity of 85, frame rate of 30 frames per second (fps), shutter speed and laser pulse duration of 100, temperature of 19.81 °C, and pH of 7.0. Post-acquisition parameters were set to a minimum brightness of 22, a maximum area of 1000 pixels, and a minimum area of 10 pixels^44^. All parameters (temperature, conductivity, electrical field, and drift measurements) were documented for quality control. After software analysis, the mean, median, and mode (indicated as diameter) sizes, as well as the concentration of the sample, were calculated, excluding outliers^44^. The number of particles per particle size curves was created using quadratic interpolation^44^.

### Oviductal EVs proteomics

Oviductal EVs (n = 3 cats, a total of 6 oviducts, in follicular phase) were pooled, frozen at −20 °C and sent to Bioproximity LLC (Chantilly, VA) on dry ice for protein extraction and proteomic analysis, according to the company’s protocols. Protein samples were prepared using a single-pot, solid phase-enhanced sample-preparation (SP3) technology^51^.

Analysis was performed using ultraperformance liquid chromatography and tandem mass spectrometry (UPLC - Thermo Easy-nLC 1200 fitted with a heated, 25 cm Easy-Spray column – MS/MS - Thermo Q-Exactive HF-X quadrupole-Orbitrap mass spectrometer). The peptide dataset (mzML format) were exported to Mascot generic format (.mgf) and searched using X!!Tandem^79^ using both the native and k-score scoring algorithms^80^, and by OMSSA^81^. RAW data files were compared with the protein sequence libraries available for the domestic cat (*Felis catus*, taxa 9685). Label free quantification (MS1-based) was used and peptide peak areas were calculated using OpenMS^82^. Proteins were required to have one or more unique peptides across the analyzed samples with *E*-value scores of 0.01 or less.

### Functional GO clustering

Data Entrez Gene IDs were mapped for all identified proteins using the R package rentrez (ver 1.2.1)^83^. The background dataset for all analyses was the cat (*Felis catus*) genome. Gene ontology (GO) analyses were performed using DAVID (https://david.ncifcrf.gov)^84^ and PANTHER (http://pantherdb.org)^85^ web-based software. For enrichment analysis, the cut off was set to *p* < 0.05. The Cytoscape 3.5.1 plugin ClueGO^86^ was used to visualize interactions of EVs proteins and networks integration, by GO terms “biological processes” and “cellular components” using the *Felis catus* genome. The evidence was set to “Inferred by Curator (IC)”, and the statistical test was set to a right-sided hypergeometrical test with a κ score of 0.7–0.9 using Bonferroni (step down). The function “GO Term fusion” was selected, the GO term restriction levels were set to 3, and a minimum of three genes or 5% genes in each GO term was used.

### Oviductal EVs labeling

Oviductal EVs (n = 3 cat oviducts) were stained by phospholipid bilayer BODIPY^®^ TR Ceramide (Invitrogen, USA). In brief, TR Ceramide stock solution (dissolved in DMSO) was added to 50 μl of total EVs (final concentration of 5 µM) and incubated for 30 minutes at room temperature. Labeled EVs were then purified to remove free dye by Exosome Spin Columns (Invitrogen, USA), following manufacturer’s instructions. Labeled EVs were immediately incubated with sperm as described below.

### Epididymal sperm isolation and incubation with oviductal EVs

Testes from domestic cats (8 months to 4 years old, n = 15) were recovered after routine neutering at local veterinary clinics and transported (at 4 °C) to the laboratory within 6 h of excision. *Corpus* and *caudal* epididymides as well as *vas deferens* were isolated and cleaned off blood vessels. Transversal dissections of the epididymis were performed and the epididymides were immersed in PBS after which the vas deferens were squeezed and sperm were allowed to swim out for 5 minutes at 38 °C. PBS containing spermatozoa was collected, centrifuged at 700 × g for 5 minutes, and supernatant was discarded. The sperm pellet was then suspended in PBS to achieve the concentration of 1 × 10^6^ sperm/mL and incubated with oviductal EVs or PBS (1 volume of sperm:2 volumes of EVs/PBS, 2.26 ± 1.03 × 10^6^ total particles) for 1 hour at 38 °C and 5% CO_2_ in air. After incubation, sperm suspension was pelleted by centrifuging at 700 x g for 5 minutes and: (1) fixed in 2% glutaraldehyde +4% paraformaldehyde in PBS and processed for transmission electron microscopy (TEM); (2) diluted to 5 × 10^6^ sperm/mL in synthetic oviductal fluid (SOF) medium and used for sperm motility analyses; (3) kept in PBS and collected at 0, 0.5, 1, 2 and 3 h for acrosome integrity analysis; or (4) processed for *in vitro* fertilization. Sperm cells were also incubated with BODIPY labeled EVs (as described above), fixed in 4% paraformaldehyde for 15 minutes and visualized by a fluorescence microscope (EVOS FL auto 2, Invitrogen, USA) with a 1,000x Plan-Apochromat oil immersion objective to evaluate EVs bound to sperm cell.

### Oviductal EVs and spermatozoa transmission electron microscopy

For transmission electron microscopy (TEM), the EVs suspensions (n = 2 cats) were fixed for 1 hour in 4% paraformaldehyde. EVs (5 μl) of each donor were dropped onto copper mesh Formvar coated carbon stabilized grids and allowed to be adsorbed to the grid for 4–5 min after which extra liquid was wicked off with filter paper^44^. A 2% Aqueous Uranyl Acetate (5 μl) solution was applied to the grid for 30 seconds (negative staining of exosomes), then whisked off with filter paper, and grids were air dried before imaging^44^. Epididymal sperm incubated with or without EVs (n = 4 cats) were fixed as described above and processed for TEM. The sperm-EVs samples preparation and imaging were performed at the Advanced Microscopy Laboratory at the University of Virginia (AMLUVA) and imaged on a Jeol JEM1230 transmission electron microscope (TEM) (40 kV) at AMLUVA.

### Sperm motility and acrosomal analyses

Sperm samples from five cats (three replicates) were incubated at 38.5 °C in SOF medium +1000 IU of Penicillin, 10 μg/ml streptomycin, 10 μg/ml of heparin, 20 µM penicillamine, 10 µM hypotaurine, 1 µM epinephrine for up to 24 h and samples collected at 0, 1, 2, 18, and 24 h for motility analysis; or in PBS with or without oviductal EVs and samples collected at 0, 0.5, 1, 2 and 3 h for acrosome integrity. For motility analysis, 10 μl of sperm samples were dropped onto a glass slide, covered with a coverslip and observed at a magnification of 200x on a positive phase-contrast microscope (Leika Leitz DM1L; Wetzlar, Germany) with a warmed (38 °C) stage. Ten fields per sample were recorded using a Nikon DS-F camera (Japan), and a minimum of 100 spermatozoa were analyzed in each sperm sample for sperm total motility (percentage of moving spermatozoa), hyperactive (star spin moving spermatozoa) and progressive (forward moving spermatozoa) motility analyses. For acrosome integrity assessment, 10 μl of sperm were fixed in 10 μl of 4% paraformaldehyde for 15 min and labeled with HOECHST33342 (for DNA) and the acrosome-specific fluorochrome fluorescein isothiocyanate-labeled peanut (Arachis hypogaea) agglutinin (FITC-PNA). At least one hundred spermatozoa were counted at 1,000x magnification (EVOS FL auto 2, Invitrogen, USA),

### *In vitro* maturation, *In vitro* fertilization and embryo culture

Ovaries from adult domestic cats were recovered after routine ovariohysterectomy at local veterinary clinics and transported (at 4 °C) to the laboratory within 6 h of excision. Cumulus cell–oocyte complexes (COCs) were mechanically isolated into HEPES (Sigma-Aldrich, USA)-buffered minimum essential medium (MEM; Life Technologies, USA) supplemented with 2 mM L-glutamine, 1 mM pyruvate (Sigma-Aldrich, USA), 100 IU/ml penicillin (Sigma-Aldrich, USA), 100 μg/ml streptomycin (Sigma-Aldrich, USA) and 4 mg/ml bovine serum albumin (BSA; Sigma-Aldrich, USA). COCs (n = 429) were incubated in 50 μl microdrops (15–25 COCs per drop) of IVM medium consisting of 1 μg/ml FSH (Vetoquinol, USA) and 1 μg/ml porcine LH (National Hormone and Pituitary Program, USA) in SAGE blastocyst medium^87^. After a 24 h *in vitro* maturation (38 °C in 5% CO_2_), COCs were inseminated with 1 × 10^6^ sperm/ml of fresh domestic cat epididymal spermatozoa incubated with (n = 231 oocytes) or without (n = 198 oocytes; Control) oviductal EVs. At 24 h post-insemination, oocytes were denuded and cleaned by gentle pipetting. Presumptive zygotes were cultured (38 °C in 5% CO_2_) in 50 μl droplets of SAGE blastocyst medium (15–25 embryos per drop) for up to 8 days. The numbers of embryos developing to 2–4 cell, (cleavage), >8 cell, and blastocyst stage were recorded on Day 2, 4 and 7 post-IVF, respectively using an inverted light microscope at a 200x magnification (Leika Leitz DM1L; Wetzlar, Germany).

In a separate study, IVF was performed as described and presumptive zygotes were collected 24 hours post fertilization and fixed in 4% paraformaldehyde. The fixed zygotes were stained with HOECHST33342 (5 μg/mL) for 30 min and visualized under a fluorescence microscope (EVOS FL auto 2, Invitrogen, USA) at a 400× magnification. The zygotes were classified as one of the two following categories: normal fertilization (presence of two pronuclei or one maternal pronuclei and one sperm head, inside the oolemma, plus the detection of two polar bodies in the perivitelline space) or polyspermic zygotes (presence of three or more pronuclei or one maternal pronucleus and multiple sperm heads, inside the oolemma plus the detection of two polar bodies in the perivitelline space). Three replicates were performed and the total number of COCs used were 68 for the control and 87 for sperm incubated with exosomes.

### Statistical analysis

The data were analyzed using IBM SPSS Statistics (version 24). A Shapiro-Wilk test was performed, and all data proved to be normally distributed. Data are presented as mean ± standard deviations. ANOVA followed by a Tukey test was used to compare EVs concentration among cats. A paired samples t-test with 95% confidence interval was used to compare motility and acrosomal integrity between samples incubated with and without exosomes. Effects of sperm-EVs incubation on polyspermy, cleavage, percentage of >8-cells and blastocyst rates (dependent variables) were analyzed by a General Linear Model (GLM) where incubation of sperm with/without oviductal EVs was the fixed factor, and the male used for fertilization was the co-variate factor.

## Supplementary information

Supplementary figure 1.

Dataset 1

Dataset 2

## Data Availability

The authors declare that all data supporting the findings of this study are available within the article, Supplementary Files, or from the corresponding author upon reasonable request. UPLC-MS/MS (mzML) file has been deposited in FIGSHARE database under 10.6084/m9.figshare.7837331 (https://figshare.com/s/85c874c0d1076a1d5894).
