# Supplementary material for: Oviductal extracellular vesicles interact with the spermatozoon’s head and mid-piece and improves its motility and fertilizing ability in the domestic cat
Source: Sci Rep. 2019 Jul 1;9:9484. doi: 10.1038/s41598-019-45857-x (PMC6603010; doi:10.1038/s41598-019-45857-x)
Supplement: Supplementary file 1 — Supplementary figure 1. [file 41598_2019_45857_MOESM1_ESM.docx]

**Supplementary files**

# Oviductal extracellular vesicles interact with the spermatozoon’s head and mid-piece and improves its motility and fertilizing ability in the domestic cat

Ferraz, M. de A. M. M.^1^*; Carothers, A. ^1^ ; Dahal, R. ^1^; Noonan, M. J.^2,3^; Songsasen, N. ^1^

^1^Center for Species Survival, Smithsonian National Zoo and Conservation Biology Institute, 1500 Remount Road, Front Royal, Virginia 22630, USA.

^2^Smithsonian National Zoo and Conservation Biology Institute, 1500 Remount Road, Front Royal, Virginia 22630, USA.

^3^Department of Biology, University of Maryland, College Park, Maryland 20742, USA.

**Supplementary Figure 1.**


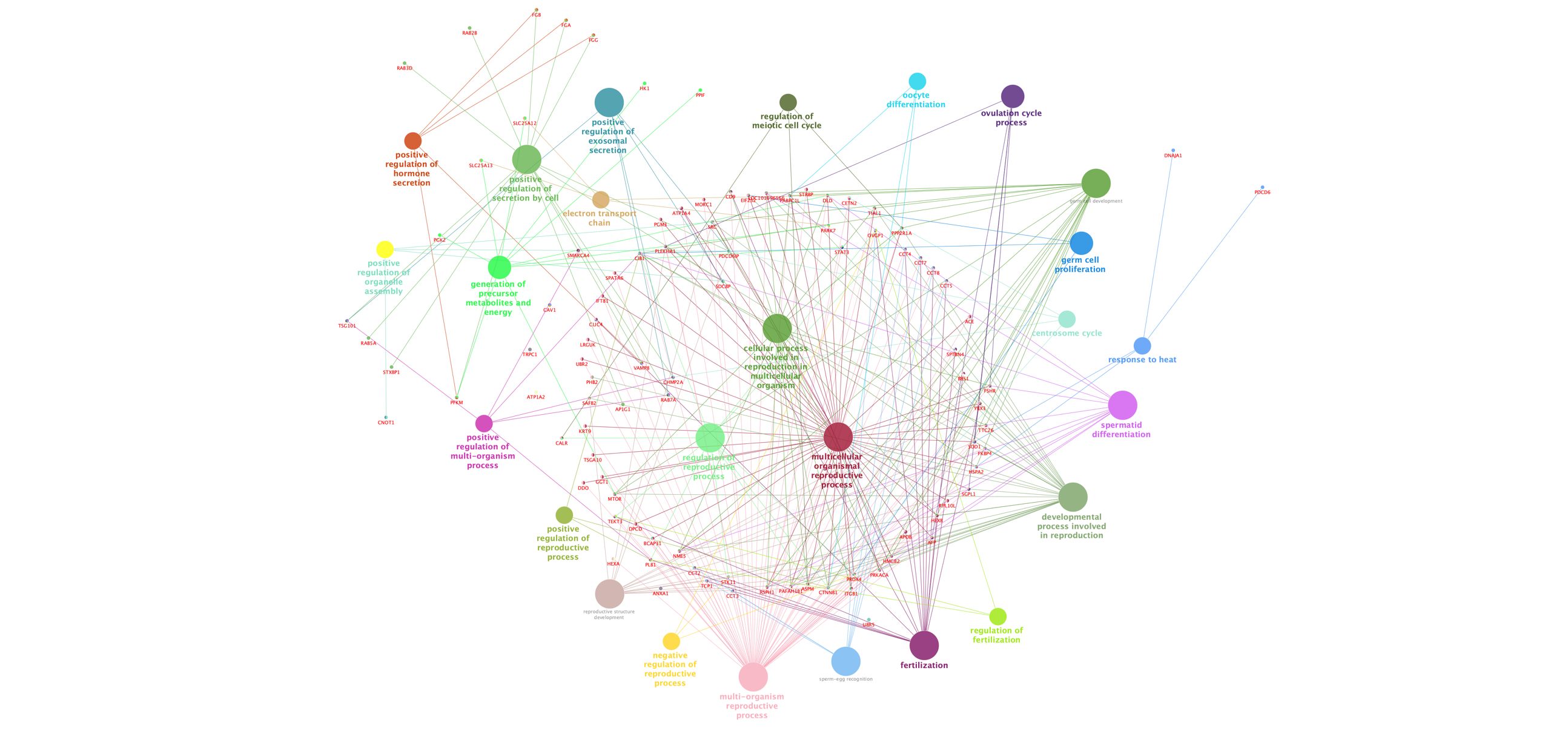


**Supplementary Fig. 1.** ﻿Functionally grouped gene ontology (GO) terms for cat oviductal exosomes proteins related to reproduction. The CytoScape plugin ClueGO was used to group the proteins into functional GO terms of “biological processes”.
